# O.4.6-11 MOVEOUT: a cluster randomised wait list design to investigate the effect of education outside the classroom on pupil’s physical activity

**DOI:** 10.1093/eurpub/ckad133.230

**Published:** 2023-09-11

**Authors:** Peter Elsborg, Lærke Mygind, Mads Bølling, Charlotte Demant Klinker, Paulina S Melby, Anne Helms Andreasen, Jan Christian Brønd, Peter Bentsen, Glen Nielsen

**Affiliations:** Center for Clinical Research and Prevention, Copenhagen University Hospital – Bispebjerg and Frederiksberg, Nordre Fasanvej 57, 2000 Frederiksberg, Denmark; Center for Clinical Research and Prevention, Copenhagen University Hospital – Bispebjerg and Frederiksberg, Nordre Fasanvej 57, 2000 Frederiksberg, Denmark; Cognitive Neuroscience Unit, School of Psychology, Deakin University, Geelong, Australia; Unit of Medical Psychology, Department of Public Health, University of Copenhagen, Øster Farimagsgade 5, 1353 Copenhagen, Denmark; Center for Clinical Research and Prevention, Copenhagen University Hospital – Bispebjerg and Frederiksberg, Nordre Fasanvej 57, 2000 Frederiksberg, Denmark; VIA University College, Research Centre for Pedagogy and Bildung, Hedeager 2, 8200 Aarhus N, Denmark; Health Promotion Research, Steno Diabetes Center Copenhagen, Borgmester Ib Juuls Vej 83, 2730 Herlev, Denmark; Center for Clinical Research and Prevention, Copenhagen University Hospital – Bispebjerg and Frederiksberg, Nordre Fasanvej 57, 2000 Frederiksberg, Denmark; Center for Clinical Research and Prevention, Copenhagen University Hospital – Bispebjerg and Frederiksberg, Nordre Fasanvej 57, 2000 Frederiksberg, Denmark; Centre of Research in Childhood Health, Department of Sports Science and Clinical Biomechanics, University of Southern Denmark, Campusvej 55, 5230 Odense, Denmark; Center for Clinical Research and Prevention, Copenhagen University Hospital – Bispebjerg and Frederiksberg, Nordre Fasanvej 57, 2000 Frederiksberg, Denmark; Department of Geoscience and Natural Resource Management, University of Copenhagen, Rolighedsvej 23, 1958 Frederiksberg, Denmark; Department of Nutrition, Exercise and Sports, University of Copenhagen, Nørre Allé 51, 2200 Copenhagen N, Denmark; peter.elsborg@regionh.dk

## Abstract

**Purpose:**

Education outside the classroom (EOtC) is a physical activity (PA)-integrating pedagogical approach that aligns PA with the primary goals of schools and is therefore thought to present an acceptable, feasible, and efficacious model for school-based PA. The aim of this study was to investigate the efficacy of an EOtC intervention that aims to increase adolescents’ (ages 10-16 years) school-based and overall PA by providing a course on the pedagogical and didactic methods of EOtC to teachers across 30 Danish schools, and subsequently the teachers implementing EOTC in their classes > 5 hours weekly over the course of one school year. We expect that pupils in the intervention group will retain higher PA intensities compared to the control group, specifically 1a) more MVPA, 1b) more LPA, 1c) less SED time. Further, we hypothesise that pupils in the intervention group will retain 2a) more running, 2b) more walking, 2c) more standing, 2d) less sitting time compared to the control group.

**Methods:**

The intervention was evaluated using a cluster randomised wait list study comparing PA behaviours amongst pupils in the intervention group with pupils in the waitlist control group.

**Results:**

In this presentation, we will present novel results from data that is currently underway. Data collection is expected to be finalised in June 2023.

**Conclusions:**

Almost 20% of Danish schools practice some form of EOtC, and the findings of this study will inform whether such practice should be further encouraged in Denmark and elsewhere, or whether alterations are required to support more pupils in meeting PA recommendations. The findings have important implications for school-based PA promotion.

Novo Nordisk Foundation (grant no. 0062703), *ClinicalTrials.gov (NCT number:* **NCT05237674).** Peter Elsborg and Lærke Mygind share first-authorship.

